# An Ethnographic Account of the British Equestrian Virtue of Bravery, and Its Implications for Equine Welfare

**DOI:** 10.3390/ani11010188

**Published:** 2021-01-14

**Authors:** Rosalie Jones McVey

**Affiliations:** Social Anthropology Department, University of Cambridge, Cambridge CB2 3RF, UK; raj47@cam.ac.uk

**Keywords:** horses, Britain, welfare, bravery, ethics, culture, ethnography, tradition, training, human–horse interaction

## Abstract

**Simple Summary:**

Bravery is an important virtue for British horse riders. This article is based on 14 months of ethnographic research, in which I spent time with horse riders (n = 35), observing their day-to-day lives and recording their riding lessons, competitions and ‘yard chatter’ in field notes and by Dictaphone. I found that when riders were fearful, they were often ridiculed, excluded and belittled. Riders’ capacity to be brave became an issue particularly when horses were thought to be defiant. Riders tried to overcome their ‘confidence issues’ by ‘getting tough’—on both themselves and on their horses—often at the demand of their instructors. When fearful riders sought alternative explanations for problematic equine behaviour (such as a veterinary diagnoses), other riders judged them as avoiding getting to grips with the ‘real issues’ (their horses’ defiance, and their own fear). Programs that aim to help riders to develop confidence without instilling a sense of ‘battle’ with the horse, and without ridiculing the rider, are likely to have positive implications on equine welfare and human safety.

**Abstract:**

This article describes the virtue of bravery in British equestrian culture and suggests that riders’ tactics for bolstering bravery may have negative implications on equine welfare. These observations are based on 14 months of ethnographic research among amateur riders and the professionals who support them (n = 35), utilising participant observation and Dictaphone recordings. Riders suffering from ‘confidence issues’ could be belittled and excluded. Instructors’ approaches towards bolstering bravery involved encouraging riders to ‘get tough’—on both themselves and on their horses. Narrative theory is employed in this article to show that riders could demonstrate their own bravery through describing the horse as defiant. Alternate narrative possibilities existed, including describing the horse as needy patient and the rider as care provider. Riders were critically aware that veterinary diagnoses could be sought or avoided in line with riders’ own dispositions. ‘Diagnoses-seeking’ behaviours could be judged negatively by others and seen as evidence of unresolved fearfulness. In conclusion, the British equestrian cultural orientation towards bravery can be associated with stressful or painful training techniques, delayed or missed diagnoses of physiological pathologies, and poor training outcomes. Programs that aim to help riders to develop confidence without instilling a sense of ‘battle’ with the horse, and without ridiculing the rider, are likely to have positive implications on equine welfare and human safety.

## 1. Introduction

First, a brief caveat. This paper utilises ethnography: a methodology of researching and writing that is underrepresented within the various disciplines that contribute to scientific understandings of animal welfare. The interdisciplinary nature of this journal and the theme of this special issue in particular provide an ideal platform for considering the affordances and limitations of this methodology for contributing to debates about domesticated equine (and more broadly, animal) welfare. While it may be unusual to read about welfare-related research that involves no control of variables, and no active experimental procedure, the advantage of the observational approach of ethnography (described in more detail in Section 2 below) is that it gives us an account of human–horse interactions as they occur within everyday settings. Ethnographic studies, such as this article, are completely qualitative in nature, involving no statistically described relationships and a more literary style of presenting findings. Studies such as these can be utilised by those pursuing quantitative methods in two ways. Firstly, ethnographic research can provide a ‘scoping study’ that identifies areas for future targeted, experimental investigation. Secondly, ethnographic research can provide a helpful complication to the understanding provided by research involving more controlled conditions and quantifiable results. This is beneficial for improving the applicability of scientific understandings of animal welfare or human–animal relationships. For example, ethnographic research is ideally positioned to aid in the design of educational programs for animal owners. Each of these aspects will be discussed further in the Section 2 and Section 4 sections below.

Equine welfare is dependent upon the knowledge and actions of those humans who carry responsibility for the provision of care, management, treatment and training. This includes owners, riders, instructors, trainers and other service providers such as veterinarians, farriers, saddle fitters, and nutritionists. The misunderstanding of equine needs and behaviours has been linked to welfare concerns [1,2]. Misunderstanding can lead to forceful or stressful training methods [3,4], and the provision of inadequate management and care regimes [5,6]. For example, Collins and colleagues find that social norms constitute one of the leading causes for compromised horse welfare in Ireland [7]. Yet among much of the literature investigating the human causes of equine welfare problems, there is a presumption that given the right sort of access to knowledge and education, owners and riders would make better welfare decisions. Recently, stakeholders invested in improving animal welfare have begun to utilise Human Behaviour Change theories, which offer a more comprehensive understanding of why, when, and how, people may adapt their behaviours (The Brooke 2017 [8], The Dog’s Trust [9]). This involves understanding people in terms of their social, economic and cultural contexts [2,10,11,12,13]. Michie’s very influential Com-B model, for example, assesses obstacles and aids to Human Behaviour Change in terms of ‘Capability’ (physical and psychological) ‘Opportunity’ (physical and social) and ‘Motivation’ (automatic and reflective) [14,15].

In this article, my aim is to describe one aspect of the ‘motivations’ that drive horse enthusiasts to behave in ways that may be detrimental to equine welfare. Michie describes ‘motivations’ as “all those brain processes that energize and direct behaviour, not just goals and conscious decision making. It includes habitual processes, emotional responding, as well as analytical decision making” [14] (p. 5). Sitting within this broad category, my contribution is to introduce the anthropological study of moral virtue as an aid to interdisciplinary understanding of the implications of human culture upon equine welfare. While equine welfare research has recently drawn on social scientific methods to understand attitudes towards welfare [10,11], anthropological frameworks for analysis and ethnographic research methods are very rarely employed (though Birke and Thompson bring ethnography into conversation with equine science when discussing welfare, [16] pp. 48–49, 117–140, and Adelman and Thompson suggest that equestrian social science ought to develop towards contributing to our understanding of equine welfare [17] pp. 267–278). Interdisciplinary contributions from this field can allow better understanding of riders’ ethical evaluations of their own and others’ conduct, by observing the ethical aspects of everyday life. This will complement, and sometimes complicate, the findings of studies that enquire about ethical perspectives or attitudes through the use of interview or survey.

The anthropological study of ethics and morality is a recently emerging field [18,19,20]. It is not the study of good and bad in the world, rather it is the study of how different people come to understand their own actions in terms of various sorts of goodness. It is therefore not committed to any particular idea of what is ‘good’ or valuable, but, as Laidlaw explains, it remains committed to the idea that *all* people are evaluative [18] (p. 3). A useful comparison for this anthropological approach is Voigt and colleagues [2], who utilise Bandura’s social cognitive theory (SCT) [21] to explain that horses can be subject to diminished welfare as a result of humans’ ‘moral disengagement’. That approach presumes that welfare is diminished by a lack of moral behaviour in certain circumstances. No doubt, that is sometimes true, but there is more complexity yet to be described in the relationship between human ethics and equine welfare. While I do not mean to suggest that all people are doing their best to be good all of the time (merely following different sorts of goodness), I do believe that we can helpfully broaden our investigation by asking what sorts of moral commitments and ethical projects may correlate with poor welfare outcomes. This entails asking what *other* moral schemes of evaluation are underway when riders interact with their horses—those ethics that are not necessarily couched in a language of welfare. This involves an awareness of moral plurality and complexity. This approach is highly complementary with Ellerman’s advice for ‘autonomy respecting’ Human Behaviour Change interventions—in which, we should take the time to understand the aims and desires of our interlocutors before attempting to educate or assist them [22]. Heleski and Anthony have drawn attention to the different sorts of moral reasoning that may inform approaches towards equestrian ethics and welfare, showing that welfare assessments are never purely scientific, but always hedged in variable schemes of moral value [23]. The present article builds on that contribution by providing ethnographic evidence that demonstrates such variable schemes of moral evaluation in practice.

Within the anthropology of ethics, virtues are understood as character traits that are held as inherently valuable, which means that they are valuable in and of themselves—not because they can achieve other goods or for utilitarian ends. Virtues are also understood as cultivated practical action. They involve not only attitudes or dispositions towards what it means to live well (such as have been studied in animal welfare science by survey-based methodologies) but also practical forms of acting in the world. We can ethnographically observe the virtues that variably guide human action as people navigate the challenges that present themselves in everyday life [18,24,25]. In this article, I will describe the virtue of bravery among British horse riders, and then discuss the implications that this virtue has on equine welfare. I will show that riders often demonstrate and cultivate their bravery through utilising narratives about equine wilfulness and defiance. In the discussion, I will suggest the welfare implications of these virtuous narratives: a higher likelihood of forceful and stressful training methods such as increased whip use; a lower likelihood of consulting veterinarians or following veterinary advice; and poorer training outcomes leading to higher risk of sale or euthanasia.

## 2. Materials and Methods

This article is informed by 14 months of ethnographic study among amateur British horse riders. Ethnographic methods involve long periods of qualitative study amongst a group of people. Ethnographers follow the lives of a relatively small number of research participants (when compared to the large population sizes required for statistical analysis) and record the idiosyncratic details of individual lives while conducting ‘participant observation’. This involves taking part in social life as it occurs, so as to form longstanding relationships with participants, giving the basis for an understanding of the depth and complexity of human society and culture. Ethnographers are increasingly completing observations of human relationships with non-human animals, within ‘multispecies ethnography’ methods [26,27]. Despite this, ethnographic methods are not frequently employed in the study of animal welfare, though Adelman and Thompson identify this as a key area for the future development of equestrian social science [17].

My research was centred around a large livery yard that was chosen for its ‘middle of the road’ status in terms of pricing, facilities, and variety of equestrian disciplines and training approaches represented. I kept a horse at this yard for the duration of study in order to have an authentic reason to meet participants daily and follow their relationships with their horses including when on horseback out on rides. From this yard, other participants were identified and recruited through networking. As well as amateur riders, I incorporated service providers into the study, and shadowed riding instructors, farriers and vets on their visits to different properties, recruiting more long-term fieldwork participants among the clients that we visited together. I came to know over 200 riders by name during the study, but consider 35 to be long-term participants. Of that 35, the average age was 38 (18–67). A total of 29 were amateur riders (28 female, 1 male) and 6 were professional instructors, horse trainers and/or yard managers (5 female, 1 male). I also studied towards BHS exams (Riding and Road safety, and Riding and Management Stage Two) at two different equestrian centres during research, utilising this as a further opportunity to observe human–horse interaction. Observations were recorded daily into a field journal. Riders’ ethical evaluations of themselves and others could be observed through their pride, guilt, gossip, blame, self-doubt, advice seeking and so on.

A Dictaphone was utilised in order to record narratives about horses verbatim. The Dictaphone was not always switched on, but was switched on for at least an hour during most days of fieldwork. Over 400 h of recordings were collected. Recordings were reviewed regularly throughout fieldwork, key themes identified, and particularly useful examples transcribed. These included both exemplars and anomalies of trends. The Dictaphone was used to record, for example, riding lessons; reports of horse behaviour immediately after rides; plans for how to approach rides when warming up at competitions; and discussions in the tack room about changes of equipment. At first, some riders were wary of their language when being recorded (censoring swear words, for example), but the long-term nature of fieldwork provides the opportunity for acclimatisation to the recording device and for trust to develop, such that riders became less likely to screen themselves for inappropriate behaviour.

This research was conducted in accordance with the declaration of Helsinki, and was granted approval by the PhD Committee of the Department of Social Anthropology (thesis: Jones McVey 2019 [28]). All human participants granted informed consent before taking part in the study, and were aware that they could cease participation at any time. I followed the ethical guidelines of the Association of Social Anthropologists (ASA) [29] and the American Anthropological Association (AAA) [30] with regards to managing consent and reducing the potential for harm on human participants (ASA: 1.4, AAA: 3-4). Participants understood that I was conducting ethnographic research on human–horse relationships in British equestrianism, and particularly that I was interested in ethics and welfare. After initial formal consent was given, consent was regularly verbally re-established with long-term participants throughout the 14 months of research, and I re-established consent on every occasion that the Dictaphone was switched on, such that no participants were recorded unknowingly. All participants have been anonymised within the account, including the names of horses and places, so that human participants remain unidentifiable. Participants were enthusiastic about the research, and keen to take part. In return, I helped with stable chores, took films for them of their competitive performances, and tried to otherwise make myself useful while observing daily interactions.

A concern raised by the long-term relationships fostered during ethnographic research is whether it is ethically acceptable not to intervene in situations that may be harmful to research participants, for the purposes of observing how those events play out. AAA and ASA guidelines emphasise the importance of non-interference within the communities being studied, and yet also recognise the development of real human relationships with accompanying responsibilities. Ethnographic researchers are inherently implicated within human relationships during fieldwork, which means that they owe their participants ethical regard and empathy. The balance between these two ethical principles (1) non-interference, and (2) ethical regard for participants, was relevant to this study when participants asked for my opinion on their plans or practices during informal interviews or observations. For the most part, I tactfully withheld my own knowledge, bouncing the question back to the rider/owner or asking who they might be able to consult for a second opinion, so that I could study knowledge practices without overly determining them. However, on occasion, I stepped in—for example, in the case of a 10-year-old girl who was bucked off her new pony several times within a week. I had got to know the family with their previous pony over several months, and the mother was desperate for advice, questioning me often as I watched, and teetering towards options that I felt were dangerous for the child. In that case, it felt unethical to withhold information that I had on their predicament (in this case, for example, overfeeding and saddle fit). Cases involving my intervention are not used as examples within this article.

Similar questions regarding the ethics of non-interference are relevant to the animals that feature within this research. Ethical guidelines relating to research utilising animals tend to work on the presumption that research involves implementing an experimental procedure, where the researcher or research team are responsible for what is done to animals within that procedure. The principles guiding ethical decisions are to ‘reduce, replace, or refine’ the use of animals in research. It is more difficult to apply these principles to ethnographic programs, where research involves observing practices already underway. It could be argued that ethnographic research contributes to the ‘reduction’ of animals employed specifically for experimental research by providing data about human–animal interaction without requiring additional animals to be put specifically to experimental use. However, this argument suggests that since the practices being observed are already underway, researchers themselves are not ethically responsible for what happens to the animals observed. AAA and ASA guidelines are yet to incorporate responsibilities towards non-humans within ethnographic studies [31] (p. 211), but multispecies ethnographers have been particularly keen to emphasise that relational networks of interaction, impact, and responsibility always occur between human researchers and animals being researched [32]. Ethical evaluation in keeping with this recognition suggests the importance of cultivating research relationships that are not harmful, and even considering enabling positive changes to occur within the process of the study.

As with human participants discussed above, there is a balance to be struck between, on the one hand, not intervening in the subject one is trying to describe, and on the other, honouring the ethical relationships and responsibilities that emerge when engaging in fieldwork. The case discussed above regarding the bucking pony is an example of a time in which I did intervene with the benefit of both species in mind, but these instances were very rare. To avoid the need to intervene, I did not aim to seek out the very worst practices of horsemanship (such as riders with terrible reputations), but to recruit ‘middle of the road’ riders and owners. I did not aim to shape riders’ or owners’ behaviours towards my own standards of equine welfare (unless there was a significant safety or welfare concern that I felt could not ethically be ignored, but this very rarely occurred), and I also did not encourage or goad cruel treatment at any time. Research ethics with animals tend to be computed on a basis of harm versus benefit. The benefit of this study is that it brings riders’ everyday moral practices into scientific dialogues regarding equine welfare within equestrian contexts. The utility of this learning outweighs the possible harms, which are best summarised as deliberately missed opportunities to improve equine welfare during interactions with owners (for the purpose of unincumbered observation), rather than any harms caused by the active implementation of an experimental process.

Ethnographic methods are limited in evidencing causational relationships, being better situated to demonstrate correlations. In this case, this means that I can observe incidents of poor welfare that may be associated with particular ethical approaches towards bravery, but I cannot assert that one distinctly causes the other. Ethnographic methods also rely upon, and benefit from, the subjective understanding of the ethnographer, and the analysis of idiosyncratic events rather than the distillation of statistically testable relationships. Nothing in ethnographic research is ‘controlled’—leading to a complexity of interacting variables that is both the strength and the weakness of this method.

Despite these limitations, this methodology provides some distinctive advantages over the quantitative-based methods that are more commonly used in efforts to understand human attitudes and behaviours within equine welfare research [6]. Surveys and targeted interviews can only tell us about respondents’ considered and deliberate responses to the particular questions chosen by the research team. For example, Visser and colleagues [33] found that riders’ assessments of equine personalities correlated with behavioural response tests (suggesting that riders can be reliable judges of equine temperament), but the investigation procedure provided riders with ten traits to assess using a numerical grading chart. Further, the horses were not previously known by the riders. These methodological features may well have worked to bring out the best potential for riders to conduct objective and careful analysis of equine temperament and behaviour. However, Horseman [9,10] suggests that British horse owners are likely to view their own horse’s behavioural and physiological requirements quite differently from the way that they think about the general horse population. In day-to-day life, riders are invested in long-term relationships with horses, are immersed within social settings, and are free to use a range of rhetorical techniques in describing their own horses to various audiences. In ethnographic observations (as we will see in the data described below), we find that horses’ temperaments are described richly, creatively, and often with a strong sense of moral meaning as part of riders’ own ethical evaluations of themselves.

Qualitative research has been utilised to understand attitudes towards equine welfare through the employment of focus groups and in-depth interviews. Horseman and colleagues used in-depth interviews to provide qualitative evidence about British equestrian attitudes towards equine welfare [9] and focus groups to investigate equestrian stakeholders’ attitudes towards welfare assessment [10]. Within those studies, the authors report that horse owners demonstrated defensive feelings towards others’ judgements about their own horse’s behaviour and welfare. It is just this sort of defensiveness and judgment that ethnographic research can further study. Yet while Horseman and colleagues’ studies directly asked participants to talk about ‘welfare’, the ethnographic approach of my research enables me to see when welfare emerges as a subject of concern within normal equestrian life, and when it does not. Ethnography enables us to notice equine welfare as it is associated with other forms of evaluation that may be the focus of riders’ judgements about themselves and others. Ethnographic research enables us to see how riders evaluate themselves and talk about their horses in day-to-day life, while actually on their horses’ back, or after being bucked off, when in conversation with instructors, or other owners, and so on. We can see how narratives change over time and are situated within contexts. We can, for example, study the sorts of narratives at play when riders actually use a whip when riding, as distinct from finding out about riders’ conscious deliberations or claims about whip use in an abstract sense [2,34,35].

## 3. Results

### 3.1. Bravery as a Virtue

Thompson and colleagues describe the British equestrian cultural relation to risk as more likely to ‘accept’ risks than to ‘mitigate’ them [36]. I would go further than that and say that, at times, some of my participants seemed to revel in the physical risk involved in handling and riding horses. This seemed to fit with a general resilience that riders held in relation to their bodies, displayed—at times—through bodily neglect. Caring for horses is hard physical work, and my equestrian friends were proud of the effects this labour had on their bodies, showing off blisters, bruises, chilblains, chapped lips and sun burn as evidence of their outside, hard-working lives and dedication to their horses. Many smoked, did not eat well, or did not eat during the day at all when with the horses, some snacking on sweets and shop-bought cakes, drinking black coffee or energy drinks to keep them going during their equestrian activities, and remarking on their lack of need for food, warmth, or other comfort, sometimes through reference to those softer types who would not be able to hack it.

My weekly email news bulletins from Horse and Hound (the oldest equestrian weekly magazine in the UK) almost always contained some dramatic injury story, foregrounding the resolve and resilience of the injured party. Michael Jung, the German Olympic Gold Medallist, and World Champion eventer, became idolised not only for his riding skill, but for the fact he won Burleigh (based in the UK, one of the world’s toughest events) in 2015 on his second horse of the day having broken his ankle that same day falling off the first ride. The extent of the injury was not understood until after the event, but this incident was reported both in magazines and in my conversation with participants with admiration, as an example of a true brave horseman, full of ‘grit’ and commitment. Birke and Brandt also report that an ethic and aesthetic of bravery was observable during their ethnographic study of British riding—they note the praise given to riders (particularly children) for their bravery when handling or riding challenging horses, and especially for remounting after a fall [37] (p. 191).

Among the 35 participants that I got to know especially well during fieldwork, there were 6 hospital visits as the result of falls during my research, and multiple minor injuries. From my observations, many accidents did not receive the hospital treatment they may have warranted, due to rider stoicism. Generally, injuries were acknowledged as part of the sport, something which cannot really be wholly avoided, and, furthermore, which one should not allow to delay or prevent one’s equestrian activities in any way. I was not at all surprised to find one participant in a thigh-high plaster cast pushing wheelbarrows, handling horses, and teaching riding, whilst limping around on crutches. A few weeks later, she was back in the saddle well before the doctor recommended it appropriate. How did I know about her doctor’s ignored advice? Because she made sure that we all knew. The doctor’s protective and prohibitive stance gave the ideal counterpoint against which she could demonstrate her own bravery and drive.

This is not to suggest that horse riders wanted to take a totally slapdash or care-free approach to managing risks. Far from it, for example, riding without a hat (riding helmet) was considered just as unprofessional, naive and incompetent as was wearing a hat for mundane, ground-based tasks. Crucially, even when deciding to avoid or limit danger (“not being completely daft” as one rider put it), the assumption was that fear should not be a feature in the rider’s decision-making practices. Instead, riders should demonstrate a proactive initiative to respond to recognised risk with pragmatism and gumption.

Despite the tangible presence of bravery as a virtue, most of my (largely amateur) participants struggled with ‘confidence issues’ at some level—whether just a flutter of nerves when they wished they could be calm during a particular riding challenge, or for a notable minority, a debilitating, highly emotional, paralysing fear that prevented them from being able to really enjoy riding much at all. Despite their anxieties, they kept on riding. ‘Horsiness’ was too deep a part of who they were. It was too painfully wrenching to consider a life without horses in it.

Particular types of horses are seen as most appropriate for nervous riders. Known as “confidence givers”, they are usually hairy, thicker set types, but crucially, have unreactive, predictable (and some say “dull”) temperaments. Many riders found the thought of riding these types of horses embarrassing and associated them with lack of skill or with a less fulfilling equestrian partnership. It was considered frustrating and humiliating for riders when their skills and confidence level did not correlate, wherein they had to ride at a ‘novice’ level (in terms of choice of horse, or level of competition) due to fear rather than lack of talent.

### 3.2. Bravery as Belonging

British equestrianism has a traditionalist character in two senses. Firstly, equestrianism is considered a quintessential example of traditional national character [37,38,39]. Secondly, equestrian skills have usually been learned in positions of apprenticeship and deference towards those with more experience, who can instil the ways in which things should be done following pre-established sensibilities [40]. Both of these aspects are related to the military history of equestrian sport in Britain. The dominant ‘modern’ equestrian sports in Britain are developments of military sports, originally designed as tests of bravery, obedience, accuracy and endurance [41]. This military history is still tangible today, for example, in the importance of concepts such as hierarchy, respect, and obedience, all of which are closely related in the British equestrian imagination to the principle subject of this article—bravery.

British equestrianism has undergone significant changes over the last thirty years. This has included the introduction of new training methods, some of which are explicitly positioned as a challenge to traditional systems [42], e.g., Roberts [43]. It has also included a broadening class demographic. While formal equestrian sports were once populated by the military elite and aristocracy, now, an increasing number of working- and middle-class amateurs take part—with the entry levels to affiliated competitions dropping as participation broadens [41,44]. Further, a market of new products and services challenge ‘old’ wisdoms and tastes, such that there are now more opportunities for a variety of styles of clothing, equipment and regime to suit different budgets and tastes (Velcro in place of buckles, animal print rugs, and so on) [39,45].

One may presume that this leads to a more inclusive setting. In fact, the destabilisation of traditional methods is accompanied by a reinvigorated sense of exclusivity, in which riders are likely to judge one another in terms of whether or not they really belong within horsey spaces, and whether or not they are conducting horsemanship in an authentic way. Specific judgements as to what might count as real, true, or proper horsemanship vary (‘traditionalists’ can find themselves critiqued by those with ‘newer’ ideas and vice versa) but there was a prevalent tendency among most riders towards, on the one hand, feeling judged by others, and on the other, actively critiquing others.

Many riders reported feeling as though they were not quite ‘horsey’ enough to feel that they really belonged within equestrian communities. This was especially likely among those who had come to horses late in life or taken a break from riding to have children or for career reasons. I witnessed riders being belittled and humiliated as ‘numpties’, ‘townies’, or ‘horse huggers’, if they were considered not properly horsey. I saw riders self-deprecating, apologising for their ineptitude, and submissive towards those who seemed more authentically rooted within equestrian spaces (because they worked within them, were highly esteemed, and/or were born into equestrian networks). Given the central importance of bravery within the military heritage and traditionalist character of equestrianism, it is perhaps unsurprising that ‘confidence issues’ could play into these dynamics of exclusivity and belittlement, such that those who were nervous when riding often felt embarrassed and ashamed, and were likely to ridicule themselves or be ridiculed by others.

### 3.3. Bravery Narratives and Defiant Horses

Ethnographic observation enables recognition of not only how bravery matters, but when bravery becomes relevant within riders’ day-to-day equestrian lives. I noticed that bravery becomes a particularly salient issue when horses are thought to be exhibiting defiant, wilful, resistant behaviour (though, academically, horses are not thought to be capable of such ‘naughtiness’ [4,46]). Riding instructors would often berate riders for ‘wimping out’ or ‘going soft’ while simultaneously interpreting the horse as deliberately challenging the riders’ leadership.

For example, in one lesson, Christine, a 64-year-old instructor of great regional acclaim, was trying to instil more bravery and authority into her pupil. She barked instructions in increasingly terrifying tones as a horse and rider approached, and then refused a jump, yet again, after multiple failed attempts. “Use your stick! I said USE IT! USE IT! YOU’RE NOT USING IT!” The rider’s eventual ‘tickle’ with the whip (as Christine called it) did not appease her—it was too little and too late. The rider was summoned over and berated. The horse had needed one good ‘reminder’ (smack) behind the saddle, exactly when Christine had asked for it, she insisted. If the rider was not going to follow her advice, she might as well get off. “You either want to jump the fence, or you don’t,” Christine pushed. “Because right now, you are no help to anybody. He’s taking the piss out of you and what are you doing about it? Nothing! Now, you ride your bloody horse or you give us all a break and go home. OK? Right then. Come again and USE YOUR WHIP when I tell you!” The rider, 26-year-old Lucy, stared down towards her horse’s neck during Christine’s barrage. She then wiped away a tear, gritted her teeth to hold back more, apologised for her incompetence, and tried again. More tears followed after the lesson, once Christine was out of earshot. Lucy explained to me why she was so upset: Her body “kept freezing” she told me, so she continued to fail to use the whip properly, ending the session thoroughly demoralised, frustrated and ashamed. She was afraid that he would ‘catleap’ over the jump, or that he was going to stop at the last minute, either way, she was afraid she was going to fall off. So, as she got towards the jump, she would just ‘bottle it’ (in her terms) which, she felt, meant that her horse was learning that he could “get away with dirty stops”. “It’s OK” she said, when I tried to offer sympathy and support after the lesson, “I need a kick up the arse when I’m riding like a wet rag. That’s why Christine is so good for me. I just hope she will agree to teach us again after that shit-show”.

Notice, the self-deprecation associated with having lost confidence, the fear of being excluded, and the desire to please the instructor. Notice, also, that the problem as Christine identifies it, is both with the ‘wimpiness’ of the rider and the defiance of the horse. These two things are tightly associated in this case. We can understand this association through narrative theory.

Narrative theory suggests that individuals make sense of their lives through stories. These stories need not be complex, long, and deliberately authored tales. They are as fleeting as the momentary recognition that makes actions and experiences morally meaningful, such as the statement “Don’t take it to heart, he is just angry because he missed the bus”. Narratives enable people to orientate themselves in relation to others [47]. Narratives utilise established plots, genres and character types that are learned within society. Genres are established ‘grammars’ for making sense of events. They can be compelling ways of understanding the world—such that people experience themselves within the world in line with their understandings of the ‘types’ of things that can happen to them. People can also understand others as types of character that are likely to impact one another in keeping with recognisable plots—in broadest terms: the hero, the villain, the lover, the trickster and so on. People use these reasonable stories as ways of understanding others’ minds—otherwise known as ‘narrative mind-reading’ [48]. These socially learned, established narrative forms do not mean that all narratives follow norms like a pre-defined series of cookie cutter replicas. Individuals can each find their own narrative understanding of events, but always utilising and re-working the available concepts and grammars of their time.

Riders were likely to make sense of their own virtues within stories about their horses. This means that even while they were ostensibly talking about their horses, and not always explicitly talking about themselves, it was quite clear how that they understood their own strengths and weaknesses in relevance to their particular understanding of their horse.

In Christine’s lesson, above, we can see that when people understand the horse within a narrative genre that identifies the equine character as defiant, that genre also suggests that the relevant character that riders ought to hold in the plot is brave, authoritative, and unyielding. The narrative link between brave riders and defiant horses can help to explain why instructors so frequently talk about the horse’s defiance as a way to rouse riders ‘grit’ and bravery—for example, “He is laughing at you! Aren’t you going to do anything?” or “I know that you are nervous, but don’t you dare let him win!”. Overcoming fear and overcoming the horse’s will are often associated projects in British equestrian culture. Riders who do not remount after a fall are seen as both letting their own fear get the better of them and letting the horse get the better of them too.

The tight association between defiant horses and the virtue of bravery also means that if riders want to demonstrate their own bravery to other riders, a great way to do that is to talk about their horse as though it were defiant. For example, consider Lesley and Tots’ case: Lesley was fiercely committed to self-improvement and to competitive success with Tots, though she almost sold him after the first few months of owning him as his behaviour and her confidence deteriorated in parallel with one another. Often she would dismount (get off the horse) in angry tears midway through a ride while unable to control Tots, and would then be very frustrated at herself, particularly since she had been proud to once identify as a brave, tough, adventurous kid. One evening, questioning whether they were ever going to get along with one another, she told me that each time she became afraid of Tots, she felt that she had lost something of herself.

However, with the help of the right instructor, Lesley was able to get Tots and her nerves simultaneously under control. This narrative is taken from a couple of months after the turning point in their relationship. I had spotted Lesley settling Tots into his stable having just returned from a competition, and I had asked whether I could turn the Dictaphone on and find out how she had got on. Lesley was keen to oblige, and spoke with a loud, cheerful, brashness. I could sense that she was proud of having completed a competition day without her instructor on hand to support, and also proud of how much less of a problem her nerves were of late:

“So, in his first [dressage] test everything was going quite well we were probably three quarters of the way through and we had to do a circle followed by a change of diagonal and I felt like he was pulling through my hands, he has always been really hard to have a contact and just doesn’t like to be told and now he’s trying to run through his forehand because he is trying to evade the contact again and I was holding him up and I was getting really fed up feeling like I was having to do all the work so as I turned the corner I really put my leg on and said, “You WILL carry me and we are going to do this!”—and he basically told me to knob off! Stuck his head in the air like a giraffe and flitted across the arena so someone thought we were going to collide with the arena so we have this funny picture of him bunny hoping across the arena but within 5 min I had him back in an outline back under control and we finished the last couple of moves on the test, but yeah he basically just told me to “do one!”.

Lesley does not explicitly talk about her own bravery, and yet her bravery is evident in the genre of the plot. We can see a clear temporal structure to the plot—there is a beginning (everything was going fine), and then a middle, in which there is a ‘jeopardy’ which ‘calls legitimacy into question’. These are elements Jerome Bruner identifies as central to the making of an autobiographical plot [49,50], drawing on Kenneth Burke [51]. The legitimacy questioned here is Lesley’s influence over Tots, which is called into question by his lazy and then defiant behaviour. However, there is a happy ending—peace is restored by the steeliness of her resolve. Lesley actually jumps temporally backwards after introducing the scene, to give us more of a back story about Tots’ character. She establishes him as evasive (“he just doesn’t like to be told what to do”) in order to bring us along on the same shared script, so that when we get to his resistance, we are ready to see it as both expectable and ridiculous rather than serious or legitimate.

We are given three visual perspectives on Tots leaping across the arena—Lesley’s initial description; the person who thinks she will collide with the arena wall; and then the photograph of him ‘bunny hopping’. This multiple visualisation adds to the drama of the moment, almost portraying it in slow motion, so Lesley manages to communicate that this was a marked and dramatic event. However, she also conveys that it does not need to be taken seriously, it can be overcome, and it is a laughable effort (‘like a giraffe’, and ‘bunny hopping’, as well as Tots’ teenage-like attitude: ‘knob off!’, he says). If there is a moral to this story, it is a triumph narrative, in which one can meet adversity with bravery, resilience and good humour. The horse, in this moral, provides the adversity by functioning as a certain sort of natural adversary (typically, more over-buoyant and wilful rather than sinister, threatening or grave). As she finished her story with a laugh that I felt compelled to share, I affirmed the moral of the story, supporting Lesley’s reading of Tots’ defiance as ridiculous and inevitable, at the same time as bearing witness to Lesley’s own bravery and resolve.

Stories such as Lesley’s were very common. All sorts of events could be, and were, described in very similar terms: first, the horse’s natural, somewhat amiable defiance creates risk and unbalances the appropriate horse/human relatedness; subsequently, and sometimes implicitly, the rider’s bravery, resolve and good humour is called into play; and finally, the rider ultimately triumphs and is able to roll their eyes at the horse’s thwarted efforts, having ‘seen it through’ with commitment and resilience.

Defiant horses require and enable a test of a particular strain of bravery (linked with authority and triumph) that no other situation quite does. If riders consider that the very best way to handle a defiant horse is to be ‘dig deep’ and ‘kick on’ bravely, then, at the same time, it seems that the very best way to demonstrate such bravery is to ride a defiant horse (or rather, to interpret one’s horse as defiant). My suggestion is that this means that when riders are highly invested in testing or demonstrating their own bravery, they are more likely to describe horses as defiant, because it is important to them to show themselves, and other riders, that they have not been beaten by their own nerves nor by equine will. Note, this is not a lack of moral engagement with the horses. All of the riders that I am describing cared deeply about their horses, and none believed battles should be won at any cost and by any means. I am not describing amoral behaviour. I am trying to emphasise a particular framing for a moral relationship between horse and rider, in which riders bolster bravery through refusing to be beaten (by their nerves or by their horses).

### 3.4. Competing Narratives

The narrative of equine defiance and rider bravery/triumph was not the only one available to riders. In fact, there was a confusing myriad of possibilities for horse riders to make sense of their horses’ behaviours and needs. Many of these other options turn attention away from focussing on the rider’s resolve. For example, one common competing narrative genre identifies the horse as a needy, honest, and trustworthy patient, who requires physical care as opposed to behavioural modification. In that narrative, riders evaluate themselves in terms of their capacity to ‘trust the horse’ and to appropriately research and resource the right diagnoses and treatment plans. While, in reality, behavioural problems and physiological pathologies are likely to co-implicate one another [4,52,53], in British equestrian narratives, there was often a drive towards establishing whether the root problem was behavioural or physiological. The distinction was important because each is so differently associated with the sorts of judgements that will be made of the rider/owner (this pattern can also be seen in the data described by Horseman et al. [11] p. 185). Of course, it is possible for a rider to be both brave AND caring, but I often observed riders trying to work out which of these dispositions they were supposed to be embodying as though they came as an either/or. I suggest that this either/or aspect comes about because, in this context, bravery is so tightly linked with authority, resolve, and toughness.

When it comes to the alternative narrative of the care-giving owner and trustworthy horse, we can see, again, that riders evaluate themselves and understand their horses as two tightly associated projects. This means that when riders receive new information about their horses, they are also receiving new possibilities for who they would be, in relation to their horse, if they believed this new knowledge to be true. Given the number of possible ways of understanding horses available on the British equestrian scene, riders can never get a definitive answer as to why their horse behaves a certain way. All explanations can be doubted or critiqued by others. Riders have to ‘plump for’ a story that resonates as true (the phrase ‘plump for’ is inspired by anthropologist Caroline Humphrey’s notion of a ‘decision event’ [54]). When they ‘plump for’ a certain reading of their horse, they simultaneously ‘plump for’ a certain sort of person that is required of them (does that sort of horse ‘need’ a leader, a boss, a friend, a carer, and so on?). This presents riders with some problems. It can be difficult for them to know whether their intuition to recognise the horse in a particular way has more to do with their own fears, desires, dispositions and preferences than it has to do with the horse’s authentic needs. One aspect of this issue is that fearful riders worry that their own fearfulness gets in the way of their capacity to ‘read’ the horse and understand its needs.

Riders intuitions, or ‘gut instincts’, for reading their horse and knowing his/her needs were often celebrated once proved to be legitimate through hindsight. However, many participants clearly experienced difficulty in evaluating and responding to ‘pangs’ of ‘gut instinct’ that ‘something is wrong’. The problem was that riders could not be sure whether their ‘gut feeling’ was coming from a genuine connection with the horse, or whether it was coming from their own fearfulness. They did not know whether to work on the ‘alarm bells’ that were ringing by developing their own bravery and grit, pushing through with stoicism and resilience, or whether they ought to be listening carefully and thoroughly investigating the possibility that something might be ‘legitimately’ preventing the horse from behaving better.

Here is Lesley talking about Tots again, in more of an unsure moment:

“Sometimes I think he is better on the [herbal] calmer. I think that it’s the changes in the grass that make the calmer work or not work. And I think the weather, he hates the wind. But then sometimes I think it is all in my head. It’s the psychology of it. I am just worrying, because I am worrying. Maybe I am just avoiding riding in the wind because then I am scared. So then I’m gonna do it because I’m not one to be beaten. But maybe I am scared because I know, deep down, that he isn’t right, that something isn’t right. Maybe the calmer is what he needs or maybe it’s masking something more important. Or maybe it is doing nothing at all and I am seeing it how I want to see it”.

It is difficult for fearful riders to seek out new knowledge, evaluate it, and trust in its utility, because they are liable to self-doubt and often deeply concerned that any solution that draws attention away from their own fearfulness may be simply ‘making excuses’ and avoiding the real issue. This is amplified because other riders and instructors are less likely to take riders seriously when they are thought to suffer from confidence issues.

One example is the case of Naomi and Blitz. Naomi was over-horsed with Blitz, as I was told by Naomi’s friends. This was mainly due to Naomi’s confidence issues. She could not ride well enough to handle his particularly wilful nature, and she had become more afraid of him following a series of falls and near falls. According to her onlookers, while Naomi at times admitted her confidence was an issue, she did not recognise the centrality of her confidence issues in the story of Blitz’s problematic behaviour, instead believing there to be physical problems causing his escapades. Every time I met with Naomi, she offered a care-giving story which followed a similar form. Typically, it would emphasise progress, speaking from a position of hindsight, Naomi would string events together to show that, finally, they had been able to identify the root (physiological) cause, and now treatment had begun and things were looking better; his symptomatic behaviours had improved.

However, other participants did not buy it. They noted that she left out key events, for example, when his behaviour was not good despite the malady apparently receiving treatment. When the treatment du jour eventually was acknowledged as failing, Naomi would move on to another possible root cause; a new back specialist, tack changes, feeding issues, a need for calming herbs and so on. She would be able to synthesise a sensible narrative around this new explanation, as well as see an ‘improvement’ upon treatment, that others did not recognise. This constant search for the ‘real reason’ for his bad behaviour was seen by others as her inability to accept the truth of the story between them: she was afraid of him and unable to confront his wilfulness, he would be better off with a more competent, braver rider, and she would be better off with a more steady, predictable, horse.

When Naomi finally received a veterinary diagnosis of stomach ulcers for Blitz, I wondered whether the nay sayers would accept that there really had been a problem and that Naomi had been correct to continue trying to tell the story in terms of Blitz’s physiological needs, ‘plumping for’ his need for care, rather than her need for bravery. However, no such change in attitude occurred. “You can’t really tell whether or not stomach ulcers are actually painful, anyway”, other participants told me, and “loads of horses have them and don’t behave that badly”, so “the gastroscopy proves nothing”. Besides, they argued, it does not explain why he has sometimes behaved fine. Furthermore, he did not really seem to improve substantively once he received treatment, which onlookers felt proved that the ulcers never really were the cause of his behaviour (a point that Naomi explained in a different way, either as learned patterns of pain behaviour, or as evidence that his stomach was still hurting him and required further treatments and investigations). Naomi and Blitz’s case shows the possible space for creativity in narrating horses’ physiological and behavioural conditions in different ways, even when a veterinary diagnosis is present.

We could describe Naomi as an example of a ‘diagnoses-seeking’ approach. Had she not found a diagnoses of stomach ulcers, she would likely have continued looking for alternative diagnoses through consultation with different vets, internet sites, experts and specialists. A social risk with this position is that other riders may see ‘diagnoses-seeking’ riders as avoiding the real issue and looking for excuses, especially if those riders seem to struggle with confidence problems. An alternative orientation is those who consult their veterinarians, farriers, or other specialists with the underlying hope of obtaining an ‘all clear’ so that they can get on with training. These ‘all clear-seeking’ riders are more eager to identify themselves as riders and trainers rather than as care providers (note, this is not a lack of moral engagement, so much as a question of which scheme of evaluation one is invested in). ‘All clear-seeking’ riders are less likely to consider physiological problems as central to the story of what is going on with their horse, and while they may well provide treatment for their horses, it is likely to be of a more pragmatic nature—as a way to get going again, rather than a reason for pausing, stopping or drastically reconfiguring training aims.

When I observed vets visiting the stable yard, it was clear within a few moments of the appointment whether the owner was leaning towards a ‘diagnoses-seeking’ or ‘all clear-seeking’ mentality—visible in the narratives that they told the vet and the way that they processed the vets’ recommendations. Research has suggested that some riders, trainers and owners aim for competitive success, while others are more motivated by the establishment of a fulfilling relationship, and many navigate someway between these two motivations [55,56], but the distinction that I am making between ‘all clear-seeking’ and ‘diagnoses-seeking’ behaviours does not completely correlate straightforwardly with the distinction between competitive and relational aims. Some riders yearned for the ‘all clear’ so as to get on with attempting to cultivate a fulfilling relationship, while others were caught up ‘diagnoses-seeking’ as a way to explain a lack of competitive success. Vets are, in turn, invested in their own ethical dilemmas when engaging with these different owner orientations towards the medicalisation of behavioural symptoms [57].

During one lengthy discussion with Bertie (a riding instructor) while out riding, we considered the way riders interpreted their horses and recognised pain. “People who want to find a problem, will always be able to find it eventually”. He told me, “If you x-ray the whole body of any horse you will find something to pin it all on. Otherwise then you find it with a psychic, or a chiropractor. There is always something there if you want to find it. And those who can’t be bothered to get into dealing with veterinary stuff can always explain pain responses away as just behavioural”.

The relevant point here is not just that riders may project their needs onto horses—that point is so longstanding it barely needs stating. The point is that riders such as Bertie and Naomi’s friends are aware of such a risk of projection, but that this does not lead towards a reliance upon academic research or veterinary advice. Even evidence-based knowledge or expert advice is considered liable to being inappropriately ‘plumped for’, because knowledge is not judged on its own merits in a stand-alone sense, it is judged as a part of riders’ attempts to narrate the human–horse relationship. This makes the issue of providing trustable education all the more complex, because knowledge evaluation is a matter of contested legitimacy that is intertwined with other regimes of judgement such as bravery and belonging.

On that point, I would speculate that had Naomi been less frequently afraid, she would have been given more authority among her peers in plumping for the care-giving story with legitimacy. That would have seemed like an authentic—or even inspired—hunch, rather than a compulsive tactic for shying away from acknowledging her own feebleness (as her critics may describe it) in relation to Blitz’s defiance. Here again, we see how exclusionary dynamics can play into riders’ capacities to evaluate themselves and understand their horses. Riders were not considered legitimately capable of handling knowledge about their horses if they were seen to be fearful, especially if they were apparently avoiding the recognition of equine defiance and so deviating attention away from attending to their own lack of resolve. While Naomi stuck with her ‘gut’ instinct in continually seeking diagnoses, she did so against the significant obstacle of disapproval among her peers. Other riders in similar predicaments may have deferred to their peers and tried to ‘dig deep’ and ‘get tough’ (such as Lucy and Lesley), employed a braver and tougher rider to ‘ride through it’, sold a horse such as Blitz on to another home, or even had him put to sleep as ‘dangerous’.

## 4. Discussion

### 4.1. Welfare Implications

I am not suggesting that bravery, in and of itself, has problematic welfare implications. After all, while it has not been the focus of this article, it is reasonable to suggest that being confident on and around a horse is a good idea. Not least, because we are now beginning to see evidence emerging academically to support the age-old equestrian knowledge that horses respond to our emotional states [58,59,60]. It could also be argued that care-giving narratives have negative welfare implications of their own—for example, ‘diagnoses seeking’ may obscure the equine learning processes that are really underway with regards to problematic behaviour and so deny horses access to effective resolutions [61]. My aim in this article is not therefore to suggest that we should do away with trying to make riders braver. Rather, I intend to suggest that the fervent judgement of bravery in a particular strain that is linked to authority and ‘toughness’ and as a form of belonging and legitimacy in British equestrianism is unhelpful. Many riders and instructors respond to fearfulness, and try to bolster bravery, in ways that are detrimental for equine welfare (not to mention human safety).

Firstly, instructors and riders were likely to utilise narratives of equine defiance as part of an effort to overcome rider fearfulness. ‘Grit’ was cultivated in response to fear, but also, simultaneously, in relation to the horse who is imagined as defiant. When this genre was employed, riders’ concerns with bravery were often met through forceful riding—digging in the heels, pulling on the reins, using a whip or spurs for punishment and to assert the rider’s authority. When these tools are used for punishment or force, rather than as part of incremental training regimes in line with learning systems, training is likely to be more painful and stressful for the horse, more dangerous for the rider, and less successful [3,62].

Secondly, since riders were keen not to be beaten by their own nerves, they were also likely to ‘push on’ with training challenges that neither they, nor the horse, were ready for. This may seem surprising, as one may presume that nervous riders would hold back and delay. Some did, with little welfare concern arising. However, the opposite pattern was also notable, that riders’ desire to overcome their own nerves would seemingly obscure their capacity to recognise a situation that really was unsafe or too much for horse and rider to handle. Researchers have commented on the importance of gradual, incremental training for welfare, safety, and success [3,4,62]. However, some of those struggling with managing their own nerves would rush into stressful situations in a bid counter their own nervousness with ‘grit’ rather than with systematic training and thorough preparation. This included one rider taking a young horse to its first competition although riding was not going at all well at home, because the rider did not want to ‘put it off’ or ‘wimp out’. Another example included a rider who competed in a cross-country competition even though a pre-training day had gone very badly. Again, she said that she felt her nerves were the problem and she needed to ‘kick on’ and ‘get on with it’ rather than delay with further training. She was eliminated half-way around the course, the horse was struck with the whip several times when refusing water, and both the rider and horse were visibly shaking as I met with them leaving the course.

Thirdly, since diagnoses seeking can be viewed in a negative light, as an example of somebody who is ‘overthinking’ and who does not have the real grit for riding, riders may be more likely to question their own sense that ‘something is wrong’ and to ‘plump for’ a story of defiance instead. Physiological pathologies may not be recognised, or diagnoses may be delayed, when riders are liable to associate diagnoses-seeking behaviours with ‘making excuses’ and the avoidance of confidence issues. This is particularly the case when riders are placed, or have placed themselves, within positions of deferral, subservience, and self-ridicule, since it is difficult from this position to evaluate information clearly and decide upon the best course of action. Horsemen et al. [63] (p. 21) and Rioja-Lang et al. [64] (pp. 10–11) identify caregivers’ misrecognition of equine pain as a key welfare challenge facing British equestrianism at present. My findings build on this knowledge, by demonstrating narratives and judgements that inhibit caregivers’ capacity to settle on a pain-based explanation. I use the language ‘settle on’ here to emphasise the uncertainties that riders experience. It is not only a question of whether they do, or do not, *recognise* equine pain, so much as it is a matter of what it would mean for them, and about them, if they did commit to a narrative in which pain is a cause.

Fourthly, given the associations noted above with ineffective, forceful and stressful training on the one hand, and missed or delayed diagnoses on the other, an unhelpful concern with ‘pushing through’ bravery problems could instigate a higher likelihood of continued behavioural dysfunctions. This may lead to euthanasia on behavioural grounds, or sale. Horses sold due to behavioural problems can get lucky, and find themselves in a home better able to meet their behavioural and physiological needs, or they can get unlucky, and move on through a number of homes, each aggravating or instilling the issues before ‘passing the buck’ on again.

### 4.2. Making Changes

Perhaps it could be argued that the welfare concerns highlighted here are really related to the narrative of equine defiance, rather than the virtue of human bravery. However, it is important that we see that narratives about horses are always also narratives about riders. When riders think about their horses, they are also experimenting with, or investing in, a particular ethical evaluation of themselves. Narrative form ties horse and rider characters together in correspondence with each other. I call these ‘contingent characters,’ since they only make sense in relation to one another. This helps to give us a sense of the challenges involved in any attempt to instil changes in riders’ understanding of their horses. Riders who are engaged in narratives with detrimental welfare and safety implications will need to ‘plump for’ a new way of articulating what is happening with their horse, and they will need to be flexible enough to accommodate the different self-concepts that can accompany these narratives. Given that the equestrian world is a realm of judgement, critique and contested expertise, this is not easy. Riders do not just need to gain more information or gain the right information, they may also need to detach from some narratives that play a fundamental role in their own sense of belonging and identity, and that are bolstered by others in positions of influence around them. This may be particularly difficult for those who are fearful, dealing with self-doubt, and searching for legitimacy in their dealings with horses.

Ellerman suggests looking for instances where change is already occurring, rather than instilling it from the outside [22]. One example of this is services and products that aim to assist riders with their nervousness, without linking that nervousness to equine defiance, and without ridiculing the rider. There is now a growing body of approaches towards confidence coaching that treat both horse and rider with respect [65]. Those systems that enable riders to decrease anxiety without instilling a ‘get tough and kick on’ approach are likely to be of great benefit to both rider safety and equine welfare. There are grounds for more research here in investigating the positive welfare implications of such approaches.

Secondly, narratives are dialogical affairs. This means that they are never solely constructed by the speaker but are built through interactions in which the listeners can respond, encourage, affirm, or question. Sometimes, I found riders were fairly settled in their narrative sense of what was going on, and at other times, I found them to be seeking affirmation and guidance from their listeners. Sometimes, riders’ narratives would seem to sweep widely, searching for the best way to hang events together. The socially reinforcing nature of narrative understanding means that significant changes can be brought about by ‘modelling’ helpful language and not being drawn into affirming unhelpful language. There is scope for programs that educate those in positions of influence—veterinarians, instructors, elite competitors, equestrian journalists and so on—about the cultural and ethical implications of their language, rather than only educating those influencers with facts about horse brains and bodies. These approaches are not infallible. People are likely to gravitate towards those who share their own orientations and vocabularies and so a balance must be struck such that we can model helpful language without creating a new form of exclusion that makes others feel unwelcome or defensive.

Those who wish to provide useable information to equestrian audiences would do well to consider the sorts of stories that riders are already invested in, and to think about what sort of self-concept their information affords. For example, one possible obstacle for the uptake of scientific evidence within equestrian practice is that descriptions of equine behaviour in terms of stimulus and response (as in Equitation Science, for example) offer little scope for imagining a compelling, meaningful, moral correspondence between horse and rider. For example, Goodwin and colleagues state “It is important that we use learning theory to explain the behavioural modification that goes on in any training context, because relying on anthropocentric or anthropomorphic explanations, however alluring and plausible, adds an unnecessary layer of complexity and leaves some aspects of the horse’s motivation open to interpretation” [41] (p. 8). Yet, we must remember that, as anthropologist and philosopher Alisdair MacIntyre tells us, “Man is essentially a story telling animal… [who] can only answer the question ‘What am I to do?’ if I can answer the prior question ‘ Of what stories do I find myself a part?’” [66] (p. 216). Scientific behavioural descriptions and objective welfare assessments often utilise language that some would consider ‘mechanomorphic’ [67]. Regardless of accuracy or veracity, they may well not ‘stick’ with people who yearn to make moral sense of a relationship, rather than rational sense of a pattern of responses.

There are grounds for further research on these topics. It may be helpful to investigate not so much whether the information that riders have is correct, but rather what tends to happen when particular sorts of description are plumped for. It is important to study how knowledge about horses and ethical attitudes actually play out in equestrian lives in order to identify areas of concern and devise plans for effective change. In sum, we need to employ a range of interdisciplinary methods that continue to ask: what sort of possibilities for horse and human relatedness do different sorts of stories afford?

## 5. Conclusions

We can utilise anthropological analysis and ethnographic methods to better understand the complex social and cultural contexts in which equine welfare exists. The ethnographic research described in this article shows that British equestrians’ ethical evaluations of horses on the one hand, and of riders on the other, are tightly associated with one another. British horse riders’ attempts to cultivate bravery often involved the belittlement of nervous riders and the interpretation of horses as defiant creatures. This was associated with forceful riding methods and rushed training aims. The negative moral judgements placed on fearful riders also presented challenges for those riders when seeking knowledge and advice from veterinarians, because riders associated ‘diagnoses-seeking’ behaviours with a lack of bravery, and therefore with a lack of legitimacy. Programs which aim to improve confidence issues without instilling a ‘get tough’ approach are likely to have positive implications for horse welfare and human safety. There is good rationale for educating influential equestrian professionals about equestrian culture as well as equine welfare in a bid to improve the latter. Those trying to provide accessible knowledge to improve equine welfare would do well to understand the ethical commitments that riders hold within different forms of narrative understanding. Stories about horses are not surface layers that are easily discarded, but deeply entwined within riders’ sense of identity and belonging. There is scope for further interdisciplinary research investigating the welfare implications of various ethical orientations and narrative forms.

## Data Availability

The data presented in this study are available on request from the corresponding author. The data are not publicly available in order to protect the confidentiality of participants, and due to the personal and subjective nature of the data.
